# The impact of P2Y12 promoter DNA methylation on the recurrence of ischemic events in Chinese patients with ischemic cerebrovascular disease

**DOI:** 10.1038/srep34570

**Published:** 2016-09-30

**Authors:** Xin-Gang Li, Ning Ma, Bo Wang, Xiao-Qing Li, Sheng-Hui Mei, Kun Zhao, Yong-Jun Wang, Wei Li, Zhi-Gang Zhao, Shu-Sen Sun, Zhong-Rong Miao

**Affiliations:** 1 Department of Pharmacy, Beijing Tiantan Hospital, Capital Medical University, Beijing, China; 2 Precision Medicine Research Center for Neurological Disorders, Beijing Tiantan Hospital, Capital Medical University, Beijing, China; 3 Department of Interventional Neuroradiology, Beijing Tiantan Hospital, Capital Medical University, Beijing, China; 4 Department of Neurology, Shaanxi Provincial People’s Hospital, Xi’an, China; 5 College of Pharmacy, Western New England University, Springfield, Massachusetts, United States of America

## Abstract

The primary mechanism of clopidogrel resistance is still unclear. We aimed to investigate whether the methylation status of the P2Y12 promoter has effects on platelet function and clinical ischemic events. Patients with ischemic cerebrovascular disease were enrolled into our study. Venous blood samples were drawn for thrombelastograpy (TEG) and active metabolite assay. Patients were divided into a case- or control-group based on the occurrence of ischemic events during a one year follow-up. Two TEG parameters between the case and control groups were statistically significant [ADP inhibition rate (ADP%): *P* = 0.018; ADP-induced platelet-fibrin clot strength (MA_ADP_): *P* = 0.030]. The concentrations of clopidogrel active metabolite had no significant difference (*P* = 0.281). Sixteen CpG dinucleotides on P2Y12 promoter were tested. Three CpG sites (CpG11 and CpG12 + 13) showed lower methylation status, which correlated with a strong association with increased risk of clinical events. Changes of MA_ADP_ and ADP% were also associated with methylation levels of CpG 11 and CpG 12 + 13. Hypomethylation of the P2Y12 promoter is associated with a higher platelet reactivity and increased risk of ischemic events in our patients. Methylation analysis of peripheral blood samples might be a novel molecular marker to help early identification of patients at high risk for clinical ischemic events.

Stroke is the leading cause of death in China, and the second major cause of death worldwide^1^. After a stroke, about 60% of patients receive antiplatelet treatments^2^,^3^. Aspirin and clopidogrel are the most widely used antiplatelet drugs. Clopidogrel is a prodrug and a thienopyridine derivative. It binds irreversibly and specifically to the P2Y12 platelet receptor, inhibiting ADP-mediated platelet aggregation. High inter-individual variability was observed in patients taking clopidogrel^4^. The approved clopidogrel labels from US Food and Drug Administration, European Medicines Agency, Japan Pharmaceuticals and Medical Devices Agency, and Health Canada state that CYP2C19 poor metabolizers may be at an increased risk for experiencing cardiovascular events when taking the drug at the recommended dose compared to those normal CYP2C19 metabolizers. Alternative medications or treatment strategies should be considered in these patients^5^,^6^.

A genome-wide association study of clopidogrel showed that the common loss-of-function CYP2C19*2 variant is a major marker of treatment response, but could only explain about 12% of the variance^7^. Poor drug response was associated with increasing age (3.8% of variance), greater body mass index (BMI) (2.3% of variance), higher triglyceride levels (1.3% of variance) and nominally lower levels of high-density lipoprotein cholesterol (1.0% of variance)^7^. However, the major reason of clopidogrel poor response remains unclear.

DNA methylation is a crucial epigenetic marker and regulator of gene expression. Methylation typically occurs at CpG sites (cytosine-phosphate-guanine sites, where a cytosine is directly followed by a guanine in the DNA sequence) in vertebrates. Cytosine is converted to 5-methylcytosine after methylation. There is an inverse relationship between CpG methylation and transcriptional activity^8^,^9^. Hypermethylation of promoter DNA is related to transcriptional silencing of gene expression^10^,^11^, resulting in decreased protein activity.

The study of DNA methylation represents a new direction to identify undiscovered mechanisms on poor antiplatelet response. The current available information about DNA methylation related to poor clopidogrel responses is limited^12^,^13^. Methylation of the P2Y12 promoter may be one potential reason for clopidogrel poor response and its subsequent clinical ischemic events. The aim of our study is to assess the relationship between P2Y12 promoter methylation levels and clinical responses in patients with ischemic cerebrovascular disease.

## Results

### Characteristics of enrolled patients

A total of 448 patients were recruited from post-stroke patients who consented to the study, and we were able to follow up 438 patients for one year either through clinic visits or telephone calls (10 patients lost follow-ups due to wrong contact information provided). Thirty patients developed ischemic events and were categorized as the case-group. Among patients who did not experience ischemic events, 30 patients were selected as matched-controls. The P2Y12 promoter DNA methylation analyses were performed for these pair-matched 60 patients. Patients’ baseline and clinical characteristics are listed in Table 1. Given that BMI, hypertension, diabetes, hyperlipidemia, coronary artery disease, family history of stroke, prior cerebral infarction, drinking, and/or smoking may be risk factors for ischemic stroke, the baseline and clinical characteristics of enrolled patients were balanced between the case and control groups. A total of 4 patients died (vascular-related mortality: cerebral infarction or cerebral hemorrhage secondary to infarction) during one year follow-up, and they were categorized into the case group. The detailed clinical characteristics of both cases and controls can be found in the Supplementary Material.

### CpG methylation, platelet function and metabolite difference

**Figure 1:**
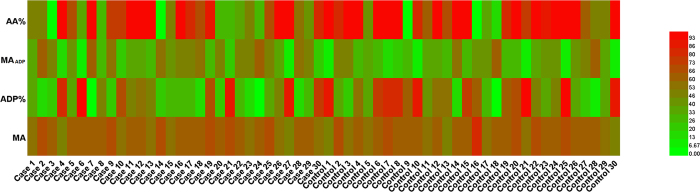
Heatmap generated from TEG parameters, MA, ADP%, MA_ADP_ and AA% values reflecting the parameter’s difference between cases and controls. Parameter values (ranged from 0–100) are displayed by a gradient color. The high data values with bright red tones and low data values with light green tones. Mixed tones depict values in-between. MA: maximum amplitude; MA_ADP_: ADP-induced platelet-fibrin clot strength; ADP%: ADP inhibition rate; AA%: AA inhibition rate.

**Figure 2:**
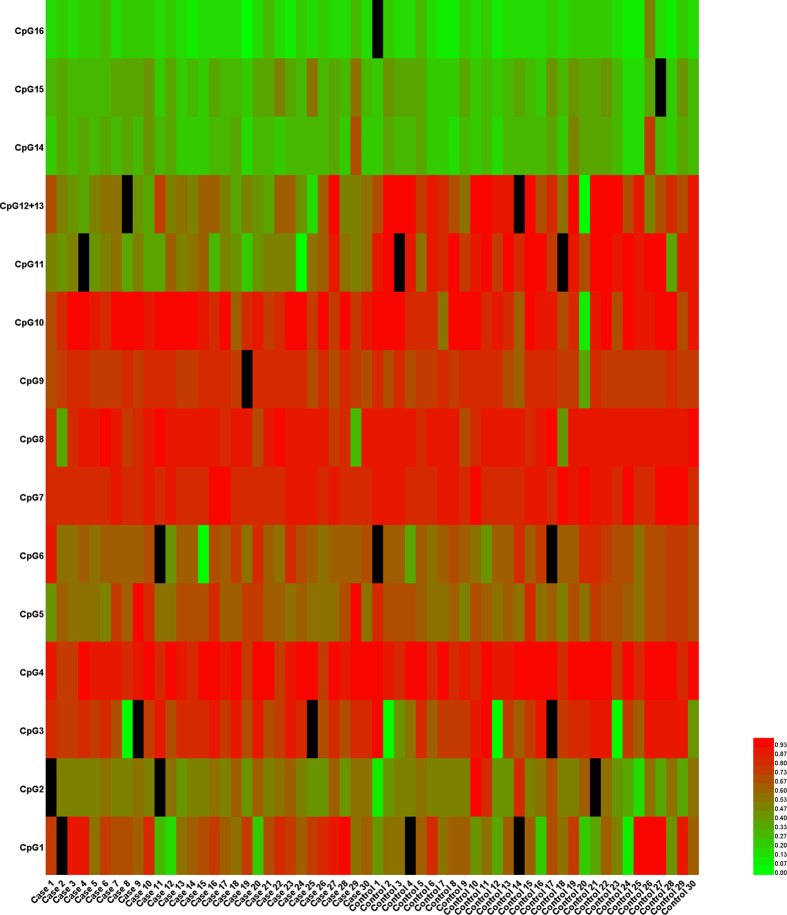
Heatmap generated from CpG methylation levels. Methylation levels are indicated by a gradient map representing the hypo- to hypermethylated status (from green to red respectively) of different CpG sites. CpG 11 and CpG 12 + 13 were significantly hypermethylated in cases as compared to controls. Several methylation levels of CpG sites cannot be tested in our study and are presented using a black color.

CpG12 and CpG13 were continuous detection sites, and Sequenom EpiTYPER technology can only measure these two sites simultaneously with the result being the average of the methylation levels at these two CpG sites. To visualize the data, we drew Heatmaps using the TEG parameters (Fig. 1) and CpG methylation levels (Fig. 2) obtained from the case group and the control-group. The scale used in the figures denotes high data values with bright red tones and low data values with light green tones. Mixed tones depict values in-between. As can be noted from Fig. 1, among the control group patients, the ADP% values have more high data values compared to the MA_ADP_ values, which have more low data values, but we could not accurately determine which parameter differed significantly between the two groups. Figure 2 is the Heatmap depicting the differences of CpG methylation levels between the two groups. It is clear that CpG11 and CpG12 + 13 in the control group have higher methylation levels (darker red indicates a higher level of methylation).

To determine if there is any significant methylation level difference for the remaining sites, a quantitative statistical method would be required. To identify predictors for developing clinical ischemic events, the differences of the metabolite concentration, TEG parameters [maximum amplitude (MA), ADP%, MA_ADP_ and arachidonic acid inhibition rate (AA%)] and methylation levels between cases and controls were analyzed using paired t-test. As shown in Table 2, the methylation levels in the three CpG sites (CpG11 and CpG12 + 13) were significantly associated with clinical ischemic events (*P* < 0.001). The differences for ADP% and MA_ADP_ in both cases and controls were statistically significant (ADP%: *P* = 0.018; MA_ADP_: *P* = 0.030). The concentrations of clopidogrel active metabolite had no significant difference (*P* = 0.281). This showed that clinical ischemic events could not be predicted by detecting drug concentration of clopidogrel.

### Relationship of active metabolite concentration and platelet function

**Figure 3:**
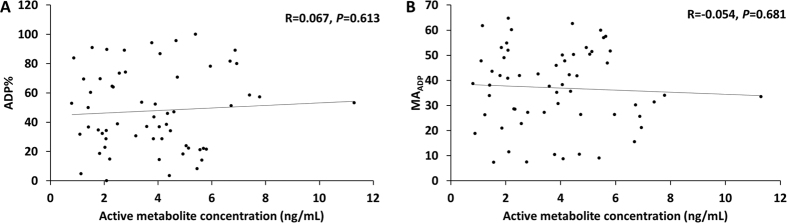
The correlation of clopidogrel active metabolite concentration and platelet activity (ADP% and MA_ADP_). The relationship was investigated by linear regression model and the lines are regression lines. R: Correlation coefficient.

Clopidogrel is metabolized to an active metabolite *in vivo* and exert its antiplatelet effect^14^. Theoretically, there should be a good correlation between the active metabolite concentration and platelet function. We made a scatter plot based on the relationships between clopidogrel activity-related TEG parameters (ADP% and MA_ADP_) and the concentrations of the active drug metabolite (Fig. 3). Figure 3 shows there is no good correlation between the two TEG parameters and metabolite concentration. The correlation coefficient R is 0.067 (*P* = 0.613) and −0.054 (*P* = 0.681), respectively. This suggests that drug exposure cannot accurately predict the anti-platelet effect of clopidogrel. There may be other factors affecting the efficacy of the drug.

### Correlation of P2Y12 methylation level and platelet function

**Figure 4:**
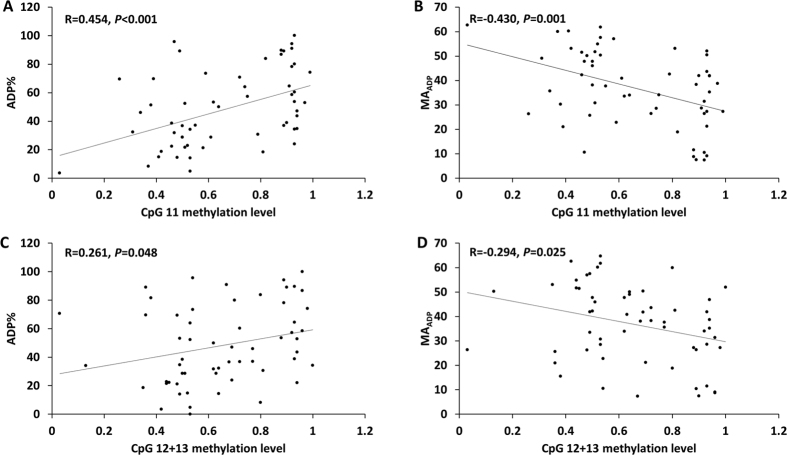
The relationship between methylation level (CpG11 and CpG12 + 13) and platelet function index (ADP% and MA_ADP_). The impact of methylation status on platelet function was assessed by linear regression model. The lines in the plots represent the regression lines. R: Correlation coefficient.

To investigate the importance of the P2Y12 gene promoter DNA methylation in platelet function, we analyzed the association of methylation levels of three CpG sites (CpG11 and CpG12 + 13) and TEG parameters (ADP% and MA_ADP_). The methylation level correlated inversely with MA_ADP_ [CpG11: R = −0.430 (*P* = 0.001); CpG12 + 13: R = −0.294 (*P* = 0.025)], and positively with ADP% [CpG11: R = 0.454 (*P* < 0.001); CpG12 + 13: R = 0.261 (*P* = 0.048)] in the linear regression model. The scatter plot of TEG parameters and methylation status is shown in Fig. 4. This correlation suggested that lower methylation level resulted in a higher residual platelet reactivity.

## Discussion

Theoretically, a causal relationship should exist within the context of drug exposure to platelet activity and to clinical events. However, our study showed that the concentration of the active clopidogrel metabolite had no significant effects on platelet activity and ultimately clinical events, suggesting that other factors might affect the final clinical outcome in our patients. We therefore focused on the role of methylation regulation on the target, P2Y12. A promoter is a region of DNA where transcription of a gene initiates. It is located near the transcription start sites of genes. In our current study, −5000 to 1000 base pairs (Chromosome 3, GRCh38.p2: 151383812–151389812) were selected to cover the P2Y12 promoter. Three regions containing rich CpG sites were selected for the methylation analysis (Table 3). A total of 60 patients (30 cases and 30 matched-controls) were selected to identify the relationship between P2Y12 promoter methylation and clinical ischemic events. Sixteen CpG dinucleotides on P2Y12 promoter were tested among the 30 cases and the 30 matched-controls. Lower methylation levels of three CpGs (CpG11 and CpG12 + 13) showed a strong association with an increased risk of clinical events. A methylation analysis of peripheral blood samples may help early identification of those patients having high risk of clinical ischemic events.

A study by Su *et al*. reported the association of methylation levels of P2Y12 promoter DNA and the risk of clopidogrel resistance in coronary artery disease patients^12^. Although many CpG dinucleotides in the P2Y12 gene promoter exist, only one fragment (GRCh37.p13: 151103600-151101600) containing two CpG dinucleotides was selected for the methylation assay. We found 11 CpG sites in this region based on the sequencing information provided, however, the detected CpG specific location was not identified in that paper. Therefore we could not make comparisons to the site locations detected in our study. The residual platelet reactivity (VerifyNow P2Y12 assay) cutoff value ≥240 reaction units indicated the existence of clopidogrel resistance^12^. The results indicated that lower P2Y12 gene promoter DNA methylation increased the risk of clopidogrel resistance in patients with albumin ≤35 g/L, current smoking, or alcohol abuse. Although clopidogrel resistance could be defined according to the platelet functions test^15^, platelet function assays are poorly standardized. Clinical ischemic events may be the gold standard for poor clopidogrel response^16^,^17^. Thus, poor or non-responders were identified during follow-up in our study. Results from this study are applicable to coronary artery disease patients with albumin ≤35 g/L, current smoking or alcohol abuse. The residual platelet reactivity for these patients can be predicted through methylation detection; however, whether the residual platelet reactivity is related to clinical events still requires validation from clinical data. In comparison, our study are for patients with an ischemic stroke, and we assess patients’ risk of clinical events by methylation assays.

It is reported that environmental and lifestyle factors could influence epigenetic mechanisms^12^. We focused on the impact of methylation on platelet function and clinical events. Matched-controls were selected according to the clinical characteristics of cases. Due to the similarity between cases and controls, we could not detect other factors such as smoking and drinking on the methylation level.

We speculated that hypomethylation of CpG11, CpG12 + 13 on the P2Y12 gene promoter may be associated with an up-regulation of P2Y12 expression. In patients with hypermethylation of the promoter, the P2Y12 receptor can be effectively inhibited by clopidogrel. In contrast to this, in patients with hypomethylation, clopidogrel cannot inhibit the P2Y12 receptor effectively, which leads to high residual platelet reactivity (MA_ADP_ and ADP%). High platelet reactivity indicated an increased recurrent risk of ischemic events. It has been reported that MA_ADP_ and ADP% can be used to predict clinical ischemic events^18^,^19^. Although our study included a small number of patients, a statistical significant difference between cases and matched-controls for MA_ADP_ and ADP% parameters (paired t-test, ADP%: *P* = 0.018, MA_ADP_: *P* = 0.030) was observed. The linear regression analysis showed that there was a correlation between CpG methylation level and MA_ADP_ and ADP% (Fig. 4).

There are several laboratory assays for testing platelet function, such as light transmission aggregometry, VerifyNow analysis, vasodilator-stimulated phosphoprotein phosphorylation assay and TEG^20^,^21^. In China, TEG is the most widely used approach^22^ due to (1) an overall assessment of *ex vivo* hemostatic function, such as thrombin, platelets, fibrin, and clotting factors; (2) using whole blood for test and processing; and (3) medical payment and reimbursement. This study aimed to observe whether methylation levels affecting the final clinical outcome. As a routine test in Chinese hospitals, we included TEG parameters in the statistical analysis. We found that methylation levels were in correlation with platelet function (ADP% and MA_ADP_) measured by TEG. But there were relatively large fluctuations of MA_ADP_ and ADP% in both case-group and matched-control group patients. Therefore deviations may exist if only TEG parameters were utilized to predict clinical events, and this may be due to the reliability of TEG platelet function test. Compared to TEG, VerifyNow P2Y12 test may be a more reliable platelet function measurement^23^,^24^,^25^. Since the VerifyNow P2Y12 test is expensive and it is not covered by insurance, it is currently not widely used in China. It is our hope that further research with the VerifyNow test will allow us to confirm the relationship between gene methylation and P2Y12 platelet activity.

Multiple genes may contribute to the clopidogrel poor response^26^,^27^,^28^. Another study found that ABCB1 promoter methylation status in whole blood appeared to be inversely associated with ABCB1 mRNA expressions and maximum platelet aggregation^13^. However, information concerning a clinical endpoint was not available. A similar study focused on ABCC3. ABCC3 gene promoter methylation correlated inversely with the gene expression, but did not seem to exhibit any impact on maximum platelet aggregation^29^. Based on the clinical ischemic events, research on the methylation status of other potential genes such as CYP2C19, CES1, and PON1 is warranted.

There are several limitations in our study: (1) P2Y12 gene expression was not measured. The association between the CpG methylation level and the quantitative gene expression remains unclear; (2) The sample size was relatively small, and our results need to be confirmed by a larger study.

In summary, the hypomethylation of P2Y12 promoter is associated with higher residual platelet reactivity and an increased risk of ischemic events. Methylation studies are a new direction to identify the mechanism of poor clopidogrel response, and research concerning the methylation status of other clopidogrel pathway genes are necessary.

## Methods

### Patient selection

The study enrolled Chinese patients with ischemic cerebrovascular disease at Beijing Tiantan Hospital, affiliated with Capital Medical University. The inclusion criteria were^30^: (1) a diagnosis of ischemic cerebrovascular disease with 70 to 99% stenosis of a major intracranial artery or an extracranial artery, confirmed by digital subtraction angiography; (2) enrollment at least five days after dual-antiplatelet therapy (aspirin 100  mg/day plus clopidogrel 75 mg/day); (3) patients received dual-antiplatelet therapy of aspirin and clopidogrel for 3 months^31^; (4) no evidence of cardioembolism, including recent myocardial infarction or atrial fibrillation within one month; and (5) age ≥30 years. Exclusion criteria were: (1) patients with atrial fibrillation, Moyamoya disease, active peptic ulcer disease, severe liver or kidney impairment, or bleeding tendency; (2) known contraindication or allergy to aspirin, clopidogrel or heparin; and (3) enrollment in another study that would conflict with this study^30^. Our study was approved by the Institutional Review Board of Beijing Tiantan Hospital (ID: KY2014-051-01), and written informed consents were obtained from patients or their close relatives. All methods were done in accordance with relevant guidelines and regulations.

### Study design

Clinical events included vascular-related mortality (death from cerebral infarction or bleeding secondary to infarction), ischemic stroke, transient ischemic attack or myocardial infarction. Ischemic stroke is a new focal neurologic deficit of sudden onset, unassociated with hemorrhage on MRI or CT, and lasting at least 24 h. transient ischemic attack is a transient episode of neurologic dysfunction caused by ischemia - either at a focal brain location, spinal cord, or retina - without acute infarction, which lasts for at least 10 min, but resolves within 24 h regardless of diffusion weighted imaging changes^18^,^30^. The occurrence of clinical ischemic events was identified during a one year follow-up visit. Patients who did not attend their follow-up visits within one year were contacted by telephone to obtain the above information.

Patients were divided into the case group if they experienced clinical events during the one year follow-up. Patients who had no recurrence of ischemic events within one year were classified as the control group. Ideally we should measure methylation levels for all patients in the control group; however, the total cost of methylation testing prohibited us to do so. We therefore selected an equal number of patients without clinical events as the control group. To best match a “case patient”, we carefully screened all control patients and selected a similarly matched “control patient” for every “case patient”. For example, the 2 pair-matched patients had same or similar characteristics in terms of age, gender, BMI, with/without clinical interventions, smoking and/or drinking status, and disease states such as hypertension, hyperlipidemia, diabetes, coronary heart disease, history of stroke, family history of stroke, etc. Due to the limited number of patients in the control group, we cannot guarantee that the paired-patients were an exact match. However, there should be no statistical significant difference between the control-group patients and case-group patients with regards to clinical features or disease states. This eliminated the interference of other factors and maximized the effect of the degree of DNA methylation on platelet function and clinical events.

### Platelet function testing

TEG is a methodology used clinically to assess platelet function and to provide a comprehensive assay of the overall clotting process. Venous blood samples were obtained from the cubital vein after five days of dual-antiplatelet treatment. The blood samples were analyzed by the TEG Hemostasis System (Haemoscope Corporation, Niles, Illinois, USA). The blood clots were linked to a stationary pin suspended in an oscillating cup containing the whole blood sample. TEG gives a quantitative analysis of platelet function based on the formation, strength, and degradation of clots in whole blood^32^. Maximal clot strength (MA_Thrombin_) is the MA, a direct function of the maximum clot strength, and it was achieved by transferring whole blood to a vial containing kaolin which was then mixed by inversion. The kaolin activated blood was transferred to a TEG cup containing CaCl_2_. To detect the fibrin contribution to clot strength (MA_Fibrin_) representing the fibrin contribution to clot strength, heparinized blood was transferred to a TEG cup containing Activator F. The contribution of P2Y12 receptor to clot formation was assessed by the addition of heparinized blood to the TEG cup along with ADP and Activator F. MA_ADP_ reflects platelet activity to ADP-induced clot strength and assesses an individual patient’s response to antiplatelet therapy. Likewise, the cyclooxygenase-1 pathway contribution to clot formation (MA_AA_) was assessed by transferring heparinized blood to AA and Activator F to the TEG cup. The ADP% and AA% were calculated as the following equations^33^.


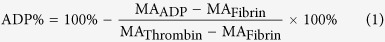



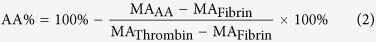


Finally, four TEG parameters, MA, MA_ADP_, ADP% and AA% were measured. All four parameters are calculated by the TEG software.

### Clopidogrel active metabolite determination

After patients took clopidogrel for at least 5 consecutive days (assuming at steady state plasma concentration), a 75 mg clopidogrel tablet was administered to each patient and 2 mL peripheral blood samples were drawn 60 min after drug administration. The samples were placed into test tubes containing 2% ethylenediaminetetraacetic acid, and 30 μL of 500 mM 2-Bromo-3′-Methoxyacetophenone protectant solution was added immediately. The tubes were vortexed for 60 seconds. After the completion of the reaction at room temperature for 10 min, the tubes were centrifuged for 5 min at 12000 rpm (10000 g). The supernatants were taken and stored in a refrigerator (−80 °C) for the analysis of the clopidogrel active metabolite concentrations. The drug concentration was analyzed using the HPLC-MS/MS assay method as reported in the literature^34^.

### Methylation analysis

Leucocytes from venous blood samples were applied to extract human genomic DNA using a commercial kit (QIAamp DNA Blood Mini Kit, Qiagen, Hilden, Germany). All DNA concentrations were greater than 500 ng/μL. The genomic DNA was treated with sodium bisulfite (EpiTect Bisulfite Kits; Qiagen). Unmethylated cytosines were converted to uracils while methylated cytosines remained unchanged^35^. Three target regions in the P2Y12 gene promoter were amplified with PCR using bisulfite-treated genomic DNAs as templates. The designed primer pairs for the three fragments are listed in Table 3. A total of 16 CpG dinucleotides in the three regions of the P2Y12 gene promoter were examined using Sequenom EpiTYPER technology (Table 3). The methylation calls were performed by the EpiTYPER software (Version 1.0, Sequenom, San Diego, CA, USA), generating methylation levels for each CpG dinucleotide or an aggregate of multiple CpG dinucleotides.

### Statistical analysis

All data analyses were performed using SPSS software (Version 17.0, SPSS Inc., Chicago, Illinois, USA). Categorical data were shown as numbers and percentages, and were analyzed using Pearson’s χ^2^ test or Fisher’s exact test (when expected value of any cell is less than 5). Continuous variables were presented as mean ± SD, and analyzed using paired t-test. A *P*-value <0.05 was considered to be statistically significant. A linear regression analysis was used to assess correlations between P2Y12 promoter methylation and residual platelet reactivity, and between active metabolite concentration and residual platelet reactivity. Each CpG site methylation levels and TEG parameters (MA, ADP%, MA_ADP_, AA%) for every patient were visualized in the Heatmap, and the Heatmap was produced using HemI software (Version 1.0.3.3, Heatmap Illustrator, Huazhong University of Science and Technology, Wuhan, China)^36^.

## Additional Information

**How to cite this article**: Li, X.-G. *et al*. The impact of P2Y12 promoter DNA methylation on the recurrence of ischemic events in Chinese patients with ischemic cerebrovascular disease. *Sci. Rep.* **6**, 34570; doi: 10.1038/srep34570 (2016).

## Supplementary Material

Supplementary Information

## Figures and Tables

**Table 1:** Clinical characteristics of patients with events and without events.

Variable	Case (n = 30)	Control (n = 30)	*P*-value*
**Age (year), mean ± SD^#^**	61.03 ± 7.22	60.43 ± 7.54	0.589
**Male, n (%)**	27 (90.0)	27 (90.0)	1.000
**Stenting, n (%)**	18 (60.0)	18 (60.0)	1.000
**Risk factors**			
** BMI, mean ± SD**	25.68 ± 3.35	25.98 ± 3.14	0.649
** Hypertension, n (%)**	17 (56.7)	18 (60.0)	0.793
** Diabetes, n (%)**	10 (33.3)	10 (33.3)	1.000
** Hyperlipidemia, n (%)**	10 (33.3)	9 (30.0)	0.781
** Coronary artery disease, n (%)**	3 (10.0)	3 (10.0)	1.000
** Family history of stroke, n (%)**	3 (10.0)	7 (23.3)	0.166
** Prior cerebral infarction, n (%)**	4 (13.3)	7 (23.3)	0.317
**Smoking, n (%)**			
** Never**	9 (30.0)	11 (36.7)	0.855
** Current**	14 (46.7)	13 (43.3)	
** Ex-smoker**	7 (23.3)	6 (20.0)	
**Drinking, n (%)**			
** Never**	14 (46.7)	14 (46.7)	0.102
** Social drinker**	10 (33.3)	15 (50.0)	
** Regular drinker**	6 (20.0)	1 (3.3)	
**Outcome, n (%)**			
** Death**	4 (13.3)	0	—
** Ischemic stroke**	13 (43.3)	0	—
** Coronary ischemic event**	5 (16.7)	0	—
** Transient ischemic attack**	8 (26.7)	0	—

^*^Paired t-test or χ^2^ test. ^#^SD: standard deviation.

**Table 2:** Statistical results of active metabolite concentration, TEG parameters (ADP% and MA_ADP_) and methylation levels between case and control groups.

	Mean ± SD (n)		Paired t-test *P*-value
	Cases group	Controls group	
Metabolite Conc.	3.90 ± 2.28	3.71 ± 1.91	0.281
MA	61.95 ± 5.51 (30)	62.42 ± 6.14 (30)	0.699
ADP%	41.19 ± 26.06 (30)	54.58 ± 27.04 (30)	0.018*
MA_ADP_	40.54 ± 15.40 (30)	33.47 ± 15.42 (30)	0.030*
AA%	63.27 ± 30.44 (30)	76.41 ± 30.96 (30)	0.159
CpG 1	0.68 ± 0.20 (29)	0.59 ± .23 (28)	0.113
CpG 2	0.52 ± 0.06 (28)	0.51 ± 0.18 (29)	0.946
CpG 3	0.77 ± 0.17 (28)	0.70 ± .28 (29)	0.324
CpG 4	0.91 ± 0.08 (30)	0.93 ± 0.08 (30)	0.237
CpG 5	0.65 ± 0.12 (30)	0.66 ± 0.09 (30)	0.965
CpG 6	0.62 ± 0.15 (29)	0.66 ± 0.11 (28)	0.115
CpG 7	0.87 ± 0.04 (30)	0.89 ± 0.05 (30)	0.088
CpG 8	0.84 ± 0.15 (30)	0.87 ± 0.10 (30)	0.299
CpG 9	0.79 ± 0.04 (29)	0.76 ± 0.09 (30)	0.194
CpG 10	0.88 ± 0.09 (30)	0.84 ± 0.18 (30)	0.303
CpG 11	0.48 ± 0.14 (29)	0.86 ± 0.13 (28)	<0.001*
CpG 12 + 13	0.52 ± 0.15 (29)	0.79 ± 0.21 (29)	<0.001*
CpG 14	0.31 ± 0.09 (30)	0.30 ± 0.11 (30)	0.791
CpG 15	0.33 ± 0.08 (30)	0.31 ± 0.07 (29)	0.223
CpG 16	0.18 ± 0.05 (30)	0.18 ± 0.08 (29)	0.909

**Table 3:** Sequences of primers for P2Y12 gene DNA methylation analysis and chromosome position of CpG sites.

Fragments	Group	DNA sequence	CpG ID	Chromosome position
Fragment 1	Forward primer	5′-TTTTTTTTAGGAGGTAGGGGTAGTG-3′	CpG1	151387988
	Reverse primer	5′-CCAACCAAAATATTCTCTAACTCCA-3′	CpG2	151387936
			CpG3	151387919
			CpG4	151387910
			CpG5	151387845
			CpG6	151387827
			CpG7	151387795
Fragment 2	Forward primer	5′-TATTTGGAATTTATTTGGATGTGTG-3′	CpG8	151386670
	Reverse primer	5′-AATTCAAAACCAACCTAACCAAAAT-3′	CpG9	151386646
			CpG10	151386638
			CpG11	151386483
			CpG12 + 13	151386429 + 151386429
Fragment 3	Forward primer	5′-TTTGTGTTAATTAAGGAATTTATAGGTTT-3′	CpG14	151385092
	Reverse primer	5′-TCACTACCCTAAATTTTTATCATTTCAA-3′	CpG15	151385073
			CpG16	151385049

## References

[b1] Sun H., Zou X. & Liu L. Epidemiological factors of stroke: a survey of the current status in china. J Stroke 15, 109–114, 10.5853/jos.2013.15.2.109 (2013). 24324946 PMC3779665

[b2] Markus H. S. Stroke genetics: prospects for personalized medicine. BMC Med 10, 113, 10.1186/1741-7015-10-113 (2012). 23016624 PMC3521189

[b3] Reny J. L. . Antiplatelet drug response status does not predict recurrent ischemic events in stable cardiovascular patients: results of the Antiplatelet Drug Resistances and Ischemic Events study. Circulation 125, 3201–3210, 10.1161/CIRCULATIONAHA.111.085464 (2012). 22615340

[b4] Greer D. M. Aspirin and antiplatelet agent resistance: implications for prevention of secondary stroke. CNS Drugs 24, 1027–1040, 10.2165/11539160-000000000-00000 (2010). 20932071

[b5] Scott S. A. . Clinical Pharmacogenetics Implementation Consortium guidelines for CYP2C19 genotype and clopidogrel therapy: 2013 update. Clin Pharmacol Ther 94, 317–323, 10.1038/clpt.2013.105 (2013). 23698643 PMC3748366

[b6] Swen J. J. . Pharmacogenetics: from bench to byte--an update of guidelines. Clin Pharmacol Ther 89, 662–673, 10.1038/clpt.2011.34 (2011). 21412232

[b7] Shuldiner A. R. . Association of cytochrome P450 2C19 genotype with the antiplatelet effect and clinical efficacy of clopidogrel therapy. JAMA 302, 849–857, 10.1001/jama.2009.1232 (2009). 19706858 PMC3641569

[b8] Venza M. . DNA methylation-induced E-cadherin silencing is correlated with the clinicopathological features of melanoma. Oncol Rep, 10.3892/or.2016.4618 (2016). 26883095

[b9] Joo J. E. . Variable promoter methylation contributes to differential expression of key genes in human placenta-derived venous and arterial endothelial cells. BMC Genomics 14, 475, 10.1186/1471-2164-14-475 (2013). 23855827 PMC3729658

[b10] Dozmorov M. G. Polycomb repressive complex 2 epigenomic signature defines age-associated hypermethylation and gene expression changes. Epigenetics 10, 484–495, 10.1080/15592294.2015.1040619 (2015). 25880792 PMC4623031

[b11] Peng R. . Promoter hypermethylation of let-7a-3 is relevant to its down-expression in diabetic nephropathy by targeting UHRF1. Gene 570, 57–63, 10.1016/j.gene.2015.05.073 (2015). 26049093

[b12] Su J. . Association of P2Y12 gene promoter DNA methylation with the risk of clopidogrel resistance in coronary artery disease patients. Biomed Res Int 2014, 450814, 10.1155/2014/450814 (2014). 24745016 PMC3976931

[b13] Yang J. . ABCB1 hypomethylation is associated with decreased antiplatelet effects of clopidogrel in Chinese ischemic stroke patients. Pharmazie 70, 97–102, doi: (2015). 25997249

[b14] Savi P. . Identification and biological activity of the active metabolite of clopidogrel. Thromb Haemost 84, 891–896, doi: (2000). 11127873

[b15] Marcucci R. . Cardiovascular death and nonfatal myocardial infarction in acute coronary syndrome patients receiving coronary stenting are predicted by residual platelet reactivity to ADP detected by a point-of-care assay: a 12-month follow-up. Circulation 119, 237–242, 10.1161/CIRCULATIONAHA.108.812636 (2009). 19118249

[b16] Nguyen T. A., Diodati J. G. & Pharand C. Resistance to clopidogrel: a review of the evidence. J Am Coll Cardiol 45, 1157–1164, 10.1016/j.jacc.2005.01.034 (2005). 15837243

[b17] Garabedian T. & Alam S. High residual platelet reactivity on clopidogrel: its significance and therapeutic challenges overcoming clopidogrel resistance. Cardiovasc Diagn Ther 3, 23–37, 10.3978/j.issn.2223-3652.2013.02.06 (2013). 24282742 PMC3839215

[b18] Wang B. . Association of thrombelastographic parameters with post-stenting ischemic events. J Neurointerv Surg, 10.1136/neurintsurg-2015-011687 (2015). 26041100

[b19] Gurbel P. A. . Adenosine diphosphate-induced platelet-fibrin clot strength: a new thrombelastographic indicator of long-term poststenting ischemic events. Am Heart J 160, 346–354, 10.1016/j.ahj.2010.05.034 (2010). 20691842 PMC2935619

[b20] Kim I. S. . Relation between the vasodilator-stimulated phosphoprotein phosphorylation assay and light transmittance aggregometry in East Asian patients after high-dose clopidogrel loading. Am Heart J 166, 95–103, 10.1016/j.ahj.2013.03.030 (2013). 23816027

[b21] Guan J. . Comparison between a new platelet count drop method PL-11, light transmission aggregometry, VerifyNow aspirin system and thromboelastography for monitoring short-term aspirin effects in healthy individuals. Platelets 26, 25–30, 10.3109/09537104.2013.865835 (2015). 24433273

[b22] Lv H. H. . Comparison of VerifyNow P2Y12 and thrombelastography for assessing clopidogrel response in stroke patients in China. Neurol Sci 37, 277–282, 10.1007/s10072-015-2407-7 (2016). 26520845

[b23] Yamaguchi Y. . Effects of VerifyNow P2Y12 test and CYP2C19*2 testing on clinical outcomes of patients with cardiovascular disease: a systematic review and meta-analysis. Platelets 24, 352–361, 10.3109/09537104.2012.700969 (2013). 22757746

[b24] Malinin A. . Validation of a VerifyNow-P2Y12 cartridge for monitoring platelet inhibition with clopidogrel. Methods Find Exp Clin Pharmacol 28, 315–322, 10.1358/mf.2006.28.5.990205 (2006). 16845449

[b25] Malinin A. . Monitoring platelet inhibition after clopidogrel with the VerifyNow-P2Y12(R) rapid analyzer: the VERIfy Thrombosis risk ASsessment (VERITAS) study. Thromb Res 119, 277–284, 10.1016/j.thromres.2006.01.019 (2007). 16563469

[b26] Calderon-Cruz B. . C3435T polymorphism of the ABCB1 gene is associated with poor clopidogrel responsiveness in a Mexican population undergoing percutaneous coronary intervention. Thromb Res 136, 894–898, 10.1016/j.thromres.2015.08.025 (2015). 26362473

[b27] Hoh B. L. . CYP2C19 and CES1 polymorphisms and efficacy of clopidogrel and aspirin dual antiplatelet therapy in patients with symptomatic intracranial atherosclerotic disease. J Neurosurg, 1–6, 10.3171/2015.6.JNS15795 (2015). PMC4915569 26587656

[b28] Li X. . PON1 Q192R genotype influences clopidogrel responsiveness by relative platelet inhibition instead of on-treatment platelet reactivity. Thromb Res 132, 444–449, 10.1016/j.thromres.2013.08.004 (2013). 23993903

[b29] Yang J. . The association of ABCC3 promoter methylation with clopidogrel response in Chinese ischemic stroke patients. Pharmazie 69, 764–768, 2014). 25985567

[b30] Li X. Q. . Association of PON1, P2Y12 and COX1 with Recurrent Ischemic Events in Patients with Extracranial or Intracranial Stenting. PLoS One 11, e0148891, 10.1371/journal.pone.0148891 (2016). 26870959 PMC4752331

[b31] Wang Y. . Clopidogrel with aspirin in acute minor stroke or transient ischemic attack. N Engl J Med 369, 11–19, 10.1056/NEJMoa1215340 (2013). 23803136

[b32] Bochsen L. . Evaluation of the TEG platelet mapping assay in blood donors. Thromb J 5, 3, 10.1186/1477-9560-5-3 (2007). 17311677 PMC1804261

[b33] Cattano D. . Perioperative assessment of platelet function by Thromboelastograph Platelet Mapping in cardiovascular patients undergoing non-cardiac surgery. J Thromb Thrombolysis 35, 23–30, 10.1007/s11239-012-0788-5 (2013). 22851059

[b34] Park J. B. . Direct measurement of active thiol metabolite levels of clopidogrel in human plasma using tris(2-carboxyethyl)phosphine as a reducing agent by LC-MS/MS. J Sep Sci 36, 2306–2314, 10.1002/jssc.201300332 (2013). 23686964

[b35] Zhang Y. . DNA methylation analysis by bisulfite conversion, cloning, and sequencing of individual clones. Methods Mol Biol 507, 177–187, 10.1007/978-1-59745-522-0_14 (2009). 18987815

[b36] Deng W. . HemI: a toolkit for illustrating heatmaps. PLoS One 9, e111988, 10.1371/journal.pone.0111988 (2014). 25372567 PMC4221433

